# EFEMP2 upregulates PD-L1 expression via EGFR/ERK1/2/c-Jun signaling to promote the invasion of ovarian cancer cells

**DOI:** 10.1186/s11658-023-00471-8

**Published:** 2023-07-07

**Authors:** Xin Shen, Xuli Jin, Shuang Fang, Jie Chen

**Affiliations:** 1 Department of Maternal and Child Health, School of Public Health, Cheeloo College of Medicine, Shandong University, Jinan, 250012 Shandong China; 2 Department of Epidemiology, School of Public Health, Cheeloo College of Medicine, Shandong University, Jinan, 250012 Shandong China; 3 Jinan Medical Center Management Committee, Jinan, 250000 China

**Keywords:** Ovarian cancer, EFEMP2, PD-L1, EGFR, ERK1/2/c-Jun pathway, Trametinib, Afatinib

## Abstract

**Background:**

Fibulin-like extracellular matrix protein 2 (EFEMP2) has been reported to be related to the progression of various cancers. We have previously reported that EFEMP2 was highly expressed in ovarian cancer and was strongly associated with poor prognosis in patients. This study intends to further explore its interacting proteins and possible downstream signaling pathways.

**Method:**

The expression of EFEMP2 was detected by RT-qPCR, ICC and western blot in 4 kinds of ovarian cancer cells with different migration and invasion ability. Cell models with strong or weak EFEMP2 expression were constructed by lentivirus transfection. The effects of the down-regulation and up-regulation of EFEMP2 on the biological behavior of ovarian cancer cells were studied through in-vitro and in-vivo functional tests. The phosphorylation pathway profiling array and KEGG database analyses identified the downstream EGFR/ERK1/2/c-Jun signaling pathway and the programmed death-1 (PD-L1) pathway enrichment. Additionally, the protein interaction between EFEMP2 and EGFR was detected by immunoprecipitation.

**Result:**

EFEMP2 was positively correlated with the invasion ability of ovarian cancer cells, its down-regulation inhibited the migrative, invasive and cloning capacity of cancer cells in vitro and suppressed the tumor proliferation and intraperitoneal diffusion in vivo, while its up-regulation did the opposite. Moreover, EFEMP2 could bind to EGFR to induce PD-L1 regulation in ovarian cancer, which was caused by the activation of EGFR/ERK1/2/c-Jun signaling. Similar to EFEMP2, PD-L1 was also highly expressed in aggressive cells and had the ability to promote the invasion and metastasis of ovarian cancer cells both in vitro and in vivo, and PD-L1 upregulation was partly caused by EFEMP2 activation. Afatinib combined with trametinib had an obvious effect of inhibiting the intraperitoneal diffusion of ovarian cancer cells, especially in the group with low expression of EFEMP2, while overexpression of PD-L1 could reverse this phenomenon.

**Conclusion:**

EFEMP2 could bind to EGFR to activate ERK1/2/c-Jun pathway and regulate PD-L1 expression, furthermore PD-L1 was extremely essential for EFEMP2 to promote ovarian cancer cells invasion and dissemination in vitro and in vivo. Targeted therapy against the source gene EFEMP2 is our future research direction, which may better inhibit the invasion and metastasis of ovarian cancer cells.

**Supplementary Information:**

The online version contains supplementary material available at 10.1186/s11658-023-00471-8.

## Introduction

By 2020, ovarian cancer is estimated to be the fifth leading cause of cancer deaths among women in the United States, accounting for 5% of all cancer deaths in women [1]. Cancer registry statistics reveal that in the past three to four decades, the mortality rates of ovarian cancer across Europe, North America, and other regions have declined by 1–2% annually [2]. However, less than half of women survive more than 5 years after diagnosis [3]. The recurrence of most ovarian cancers is due to the rapid progression of the cancer and resistance to treatment. Once cancer recurs, the interval between subsequent treatments is gradually shortened [4]. The recurrence of ovarian cancer in 75% of advanced women is incurable [5]. Therefore, elucidating the mechanism of metastasis and invasion of ovarian cancer is essential for finding new treatment strategies and improving prognosis.

Fibulin-like extracellular matrix protein 2 (EFEMP2) with epidermal growth factor is a member of the fibulin family, also known as MBP1 or fibulin-4. This protein family is composed of a tandem array of calcium-binding epidermal growth factor (EGF)-like modules and a fibulin-type C-terminal domain [6]. Early studies have shown that EFEMP2 is a product of a candidate oncogene, in mutant p53-independent or dependent mutants, it can exhibit its oncogenic activity [7]. In recent years, the expression of EFEMP2 in different types of cancers has been controversial, in that, in cancers, such as breast, lung, and endometrial cancers, EFEMP2 plays a protective role [8–12]. On the contrary, in cancers, such as ovarian cancer, glioma, osteosarcoma, and aneurysm, EFEMP2 is highly expressed, considered a candidate oncogene, and associated with poor prognosis of the tumors [13–18]. EFEMP2 has also been considered as a potential biomarker for colorectal cancer and glioma subtypes [19, 20]. Although we previously found that EFEMP2 was overexpressed in ovarian cancer and related to its poor prognosis [13], the molecular mechanism of EFEMP2 in the metastasis and invasion of ovarian cancer cells remains elusive and needs to be further explored.

Programmed death ligand 1 (PD-L1) is a secreted transmembrane protein and an important immune checkpoint protein, which can bind to programmed death 1 (PD-1) on T-lymphocytes to trigger immunosuppressive signals. T-lymphocytes play a key role in killing cancer cells, which exhibits immune escape by expressing PD-L1 [21]. PD-L1 was a key target of anticancer therapy, and its expression in tumor tissues of patients with renal cell carcinoma, esophageal carcinoma, gastric carcinoma, and ovarian cancer suggested poor prognosis [22]. The expression of PD-L1 in tumor cells was regulated by various signaling pathways, including NF-κB, MAPK, mTOR, JAK/STAT and c-Myc [23, 24]. Studies have shown that blocking the PD-1/PD-L1 pathway can enhance endogenous anti-tumor immunity by restoring the function of T lymphocytes [25]. The overexpression of PD-L1 was closely related to the activation of Epidermal growth factor receptor (EGFR). In malignant melanoma, EGFR up-regulated PD-L1 through the EGFR/STAT3 pathway [26]. EGFR activation up-regulated PD-L1 expression through the ERK1/2/c-Jun pathway in non-small cell lung cancer (NSCLC) [27], and glutamine modulated PD-L1 expression through the EGFR/ERK/c-Jun pathway in kidney cancer [28]. The up-regulation of PD-L1 in different cancer cells may occur through different mechanisms. PD-L1 or PD-1 as markers of EGFR signal suggested that EGFR mediates tumor immune escape [29]. In conclusion, the p-ERK1/2/p-c-Jun pathway might play a key role in EGFR-caused remodeling of PD-L1 expression.

We established ovarian cancer cell models with down-expression and up-expression of EFEMP2, respectively, and observed that overexpression of EFEMP2 increased the invasion ability of ovarian cancer cells, while its down-expression inhibited the invasion characteristics of ovarian cancer cells. We further identified the downstream signaling pathway of EFEMP2 and its possible effector proteins using the tumor signaling pathway chip. Overexpression of EFEMP2 up-regulated PD-L1 expression by activating EGFR/ERK1/2/c-Jun signal, while down-expression of EFEMP2 has the opposite effect. Recent researches have shown that PD-L1 plays an important role in immune escape of ovarian cancer cells, and the clinical trial results of targeted immunotherapy for PD-L1 in ovarian cancer patients have not been as dramatic as those in melanoma or non-small cell lung cancer [30, 31]. Therefore, a full understanding of the mechanism of PD-L1 in the development of ovarian cancer may be a feasible direction to improve its clinical efficacy. Currently, more and more studies have focused on the complex bidirectional regulation between EMT state and PD-L1 signal, which ultimately leads to tumor immune escape and tumor invasion [32]. In our study, both EFEMP2 and PD-L1 could promote the EMT process and increase the invasion ability of ovarian cancer cells. In PD-L1 knockout ovarian cancer cells, overexpression of EFEMP2 cannot promote EMT and cancer cell invasion and metastasis. Meanwhile, in ovarian cancer cells overexpressing PD-L1, EFEMP2 knockdown did not prevent EMT and cancer cell invasion and metastasis. PD-L1 was essential for EFEMP2 to promote the invasion and metastasis of ovarian cancer cells. This study for the first time clarified how EFEMP2 regulated PD-L1, and in the microenvironment of ovarian cancer, EFEMP2 could target PD-L1 to promote EMT process and cancer cell invasion and metastasis.

## Methods

### Cell lines and culture

Human ovarian clear cell cancer cell line ES-2 (ZQ0067) and ovarian adenocarcinoma cell line CAOV-3 (ZQ0484) were acquired from Shanghai Zhong Qiao Xin Zhou Biotechnology Co., Ltd, and adenocarcinoma cell types SKOV-3 (TCHu185) and OVCAR-3 (TCHu228) were obtained from the National Collection of Authenticated Cell Cultures, Chinese Academy of Sciences. All cells were cultured in complete medium (McCoy's 5A for ES-2 and SKOV-3, DMEM for CAOV-3, and RPMI-1640 for OVCAR-3) containing 10% fetal bovine serum (FBS, Gibco) and 1% penicillin‐streptomycin, and maintained in 37 ℃ and 5% CO_2_ incubator.

### Immunocytochemistry (ICC)

Cells were seeded at 7 × 10^4^ cells per well. After 24 h of incubation, fixed the cells with 4% paraformaldehyde for 1 h. Endogenous peroxide was first inactivated with hydrogen peroxide, and then rabbit anti-human EFEMP2 antibody (12004-1-AP, Proteintech) and PD-L1 antibody (ab213524, Abcam) were used at 4 ℃ overnight. The next day, the cells were incubated with a secondary antibody at 37 ℃ for 30 min and then stained with the DAB kit (ZSGB-Bio, China). The OLYMPUS BX63F microscope (Japan) and OLYMPUS imaging software 1.16 was used to collect immunocytochemical images.

### RT-qPCR

Total RNA in ovarian cancer cells was extracted using Trizol reagent (TaKaRa Biotechnology, China) and quantified and diluted to 500 ng/µl. Then, 2 µl of total RNA was used for reverse transcription (PrimeScript RT kit with gDNA eraser; TaKaRa Biotechnology). A 20 µl reaction system containing 10 µl Power SYBR Green PCR master mix (TaKaRa Biotechnology), 6.4 µl DNAse/RNAse-free water (Sigma‐Aldrich), 0.8 µl of each primer, and 2 µl cDNA was prepared and RT-qPCR was performed in triplicate using the LightCycler 480 System (Applied Biosystems, Inc; Thermo Fisher Scientific, Inc). The mRNA expression levels of target genes were analyzed according to Formula 2^−ΔΔCt^. Design and synthesis of the primers for internal reference (β-actin) and target genes were completed by TaKaRa Bioengineering Co., LTD., and the primer sequences were as follows: EFEMP2-F: 5′-GCTGCTACTGTTGCTCTTGGG-3′, EFEMP2-R: 5′-GGGATGGTCAGACACTCGTTG-3′; PD-L1-F: 5′-GGTAAGACCACCACCACCAAT-3′, PD-L1-R: 5′-TGATTCTCAGTGTGCTGGTCAC-3′.

### Protein isolation and western blot

The cells were lysed with RIPA lysis buffer. BCA protein quantification kit (BOSTER, China) was used to measure its concentration. The same amount of protein samples in each lane was separated (10% SDS-PAGE), and the separated proteins were transferred onto PVDF membranes and then blocked for 1 h. Subsequently, the membranes were incubated with the primary antibody (EFEMP2 12004-1-AP, Proteintech; PD-L1 ab213524, Abcam; Phospho-EGFR (Ser1070) AF3044, Affinity; Phospho-EGFR (Tyr1068) #3777, EGFR #4267, Phospho-SAPK/JNK (Thr183/Tyr185) #4668, SAPK/JNK #9252, Phospho-p44/42 MAPK (Erk1/2) (Thr202/Tyr204) #4370, p44/42 MAPK (Erk1/2) #4695, c-Jun #9165, Phospho-c-Jun (Ser73) #3270, Epithelial-Mesenchymal Transition (EMT) Sampler Kit #9782, Cell Signaling) overnight. The blots were incubated in corresponding secondary antibodies for 1 h the next day and developed (Pierce ECL Western blot analysis substrate; Thermo Fisher Scientific, Inc.).

### Migration and invasion assays

Cells migration and invasion abilities were detected by the transwell chambers, which were prepared in advance with tiled Matrigel (invasion assay) or untiled Matrigel (migration assay). The same volume of cells was seeded in each upper chamber (200 μl, approximately 2 × 10^5^ cells) and 800 μl medium containing 20% FBS was added to the lower chamber. After incubating at 37 ℃ for 24 h, wiped the cells that had not passed through the chamber, then the penetrating cells were fixed with 4% paraformaldehyde for 30 min, stained with haematoxylin, and counted under the microscope (OLYMPUS BX63F, Japan). The number of cells passing through the chamber represented the ability to migrate or invade.

### The plate clone formation assay

The cells were seeded in 6-well plates with 500 cells per well. The plates were incubated for 10 d, washed thrice with PBS, stained with crystal violet for 10 min, rinsed off the excess dye with tap water slowly, and finally dehydrated with anhydrous ethanol. Under an inverted microscope, clusters of 50 or more cells were considered to be a colony. The colony number was expressed as mean ± standard deviation (mean ± SD).

### Cell transfection and stable cell line construction

Lentiviruses harboring vectors were used to generate stable cell lines. Shanghai Genechem Co., Ltd was the designer and supplier of the EFEMP2 RNAi (Target Seq: ccACCATTGAAGAGGTTGATT and ctTCAACTCCTATGGGACCTT) and PD-L1 RNAi (Target Seq: gaCCTATATGTGGTAGAGTAT) viruses and LV-EFEMP2 and LV-PD-L1 over-expressed viruses used in this experiment. A certain amount of virus was added into the cell culture medium and cultured for 72 h, fluorescence was observed using the imager (ImageXpress Micro Confocal, Molecular Devices). Furthermore, western blot, RT‐qPCR and ICC were performed to verify the transfection effect.

### Establishment of subcutaneous graft tumor and ascites tumor models in nude mice

BALB/C‐nu/nu (nude) mice were purchased from the National Resource Center for Rodent Laboratory Animal of China. For the subcutaneous graft tumor model, 5 nude mice in each group were injected subcutaneously with 1.0 × 10^7^ cells, and the tumor volume was measured weekly. After eight weeks of routine monitoring, mice were sacrificed by carbon dioxide euthanasia and tumors were peeled out. The calculation formula for tumor volume is: length × width^2^ × 0.5, and expressed as mean ± SD. For the ascites tumor model, 12 nude mice in each group were injected intraperitoneally with 1.5 × 10^7^ cells. After 10 days of routine feeding, nude mice in each group were randomly divided into 4 treatment groups with 3 mice in each group. Normal saline 100 µl, afatinib (3 mg/kg/day) 100 µl, trametinib (2 mg/kg/day) 100 µl, afatinib (3 mg/kg/day) 100 µl + trametinib (2 mg/kg/day) 100 µl were injected intraperitoneally for 7 consecutive days, respectively. After anesthetizing mice with 10% chloral hydrate (0.3 ml/100 g), abdominal tumor spread in nude mice was observed using IVIS Spectrum imaging (PerkinElmer). Animal experiments were approved by the Animal Protection and Use Committee of Shandong University and complied with relevant national regulations.

### Phosphorylation pathway profiling array

The phosphorylation pathway profiling array was processed using the Human Phosphorylation Pathway Profiling Array C55 kit (RayBiotech, Inc.), following the manufacturer's instructions. Cells were lysed on ice, and a BCA protein quantification kit (BOSTER, China) was used to measure the protein concentration. Subsequently, the membranes (RayBiotech) were immersed in the blocking buffer for 1 h, added with the sample diluted with the blocking buffer at 4 ℃. The next day, membranes were washed and incubated with biotin-conjugated anti-cytokine, and then incubated with 1000× HRP-anti-rabbit IgG concentrate. The membranes were scanned with ImageQuant LAS4000 Scanner, then normalization was performed with positive control spots (limma, R package).

### KEGG pathway enrichment analysis

The KEGG (http://www.kegg.jp/) database assigned functional significance to genes and genomes at the molecular and higher levels. R package (clusterProfiler) was used for KEGG pathway enrichment analysis of differentially expressed genes and then Fisher's exact test was employed. The selection criteria were that the number of different proteins was not less than two and *P* value < 0.05 was considered statistically significant.

### Co-immunoprecipitation (co-IP)

First, the total protein was extracted and its concentration was determined. Total protein 10 μl was taken as input, and the remaining samples were added to the corresponding antibody and shaken overnight at 4 ℃. Then, 10 μl protein A agarose beads were added into the protein extract and incubated at 4 ℃ for 3 h. After the immunoprecipitation reaction, agarose beads were centrifuged to the bottom of the tube at 3000 rpm (revolutions per minute) for 3 min, the supernatant was carefully sucked off and the agarose beads were washed with 1 ml lysis buffer for 3–4 times. Finally, 15 μl of 2 × SDS loading buffer was added and boiled for 5 min. The interacting protein was analyzed by western blot at last.

### Statistical analyses

Continuous variables were expressed as mean ± SD and categorical variables were expressed as n (%). Two-tailed Student’s *t*-test and One-Way ANOVA [SNK (Student–Newman–Keuls) or Dunnett-*t*-test between groups] were used to compare continuous variables of two groups and multiple groups, respectively. The Chi-square test was used for categorical variables. SPSS (Version 23.0) and R (limma package, clusterProfiler package) were used for statistical analysis. The statistical graph was generated with GraphPad Prism 8.0.2. *P* < 0.05 was considered statistically significant.

## Results

### EFEMP2 is positively correlated with the invasion ability of ovarian cancer cells

**Fig. 1:**
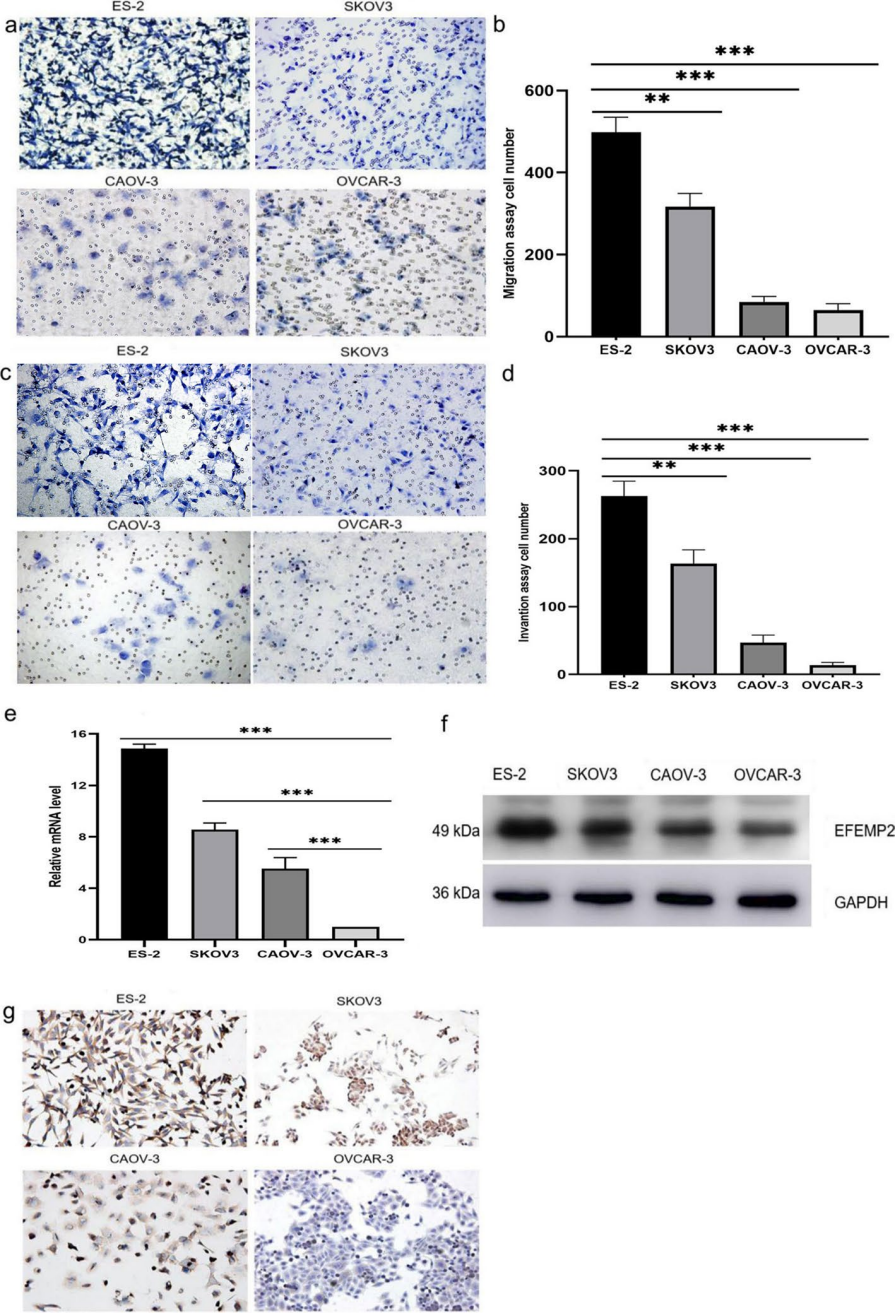
The expression of EFEMP2 in ES-2, SKOV-3, CAOV-3, and OVCAR-3 cells and its relationship with migration and invasion abilities. **a** ES-2, SKOV-3, CAOV-3, and OVCAR-3 cells migration ability was tested with a transwell chamber without Matrigel, and (**b**) the histogram demonstrated the number of cells passing through the chamber membrane. **c** The transwell chamber covered with Matrigel detected the invasion ability of ES-2, SKOV-3, CAOV-3, and OVCAR-3 cells, and (**d**) the histogram demonstrated the number of cells passing through the chamber membrane coated with Matrigel. **e** EFEMP2 mRNA levels in the four ovarian cancer cell lines were detected by RT-qPCR. Protein expression of EFEMP2 in the four ovarian cancer cell lines was detected by western blot (**f**) and ICC (**g**). Magnification × 200, *p < 0.05, **p < 0.01, or***P < 0.001

Transwell chambers were used to detect the migration and invasion abilities of four different ovarian cancer cell lines (ES-2, SKOV-3, CAOV-3, and OVCAR-3). Figure 1a, b and Additional file 1: Table S1 showed the migration ability, while Fig. 1c, d and Additional file 1: Table S2 showed the invasion ability of the cell lines. ES-2 had the strongest migration and invasion ability, followed by SKOV-3, CAOV-3 and OVCAR-3. RT-qPCR was used to detect the mRNA expression of EFEMP2 (Fig. 1e and Additional file 1: Table S3) while western blot and ICC were performed to examine EFEMP2 protein expression (Fig. 1f, g) in ovarian cancer cell lines. These three experiments showed an identical trend; the mRNA and protein expression levels of EFEMP2 in ES-2 cells were the highest, followed by SKOV-3, CAOV-3, and OVCAR-3. The expression trend of EFEMP2 was consistent with the migration and invasion ability of these four cells. EFEMP2 was positively related to the migration and invasion ability of ovarian cancer cells.

### Establishment of EFEMP2 stable down- and over-expression cell models

**Fig. 2:**
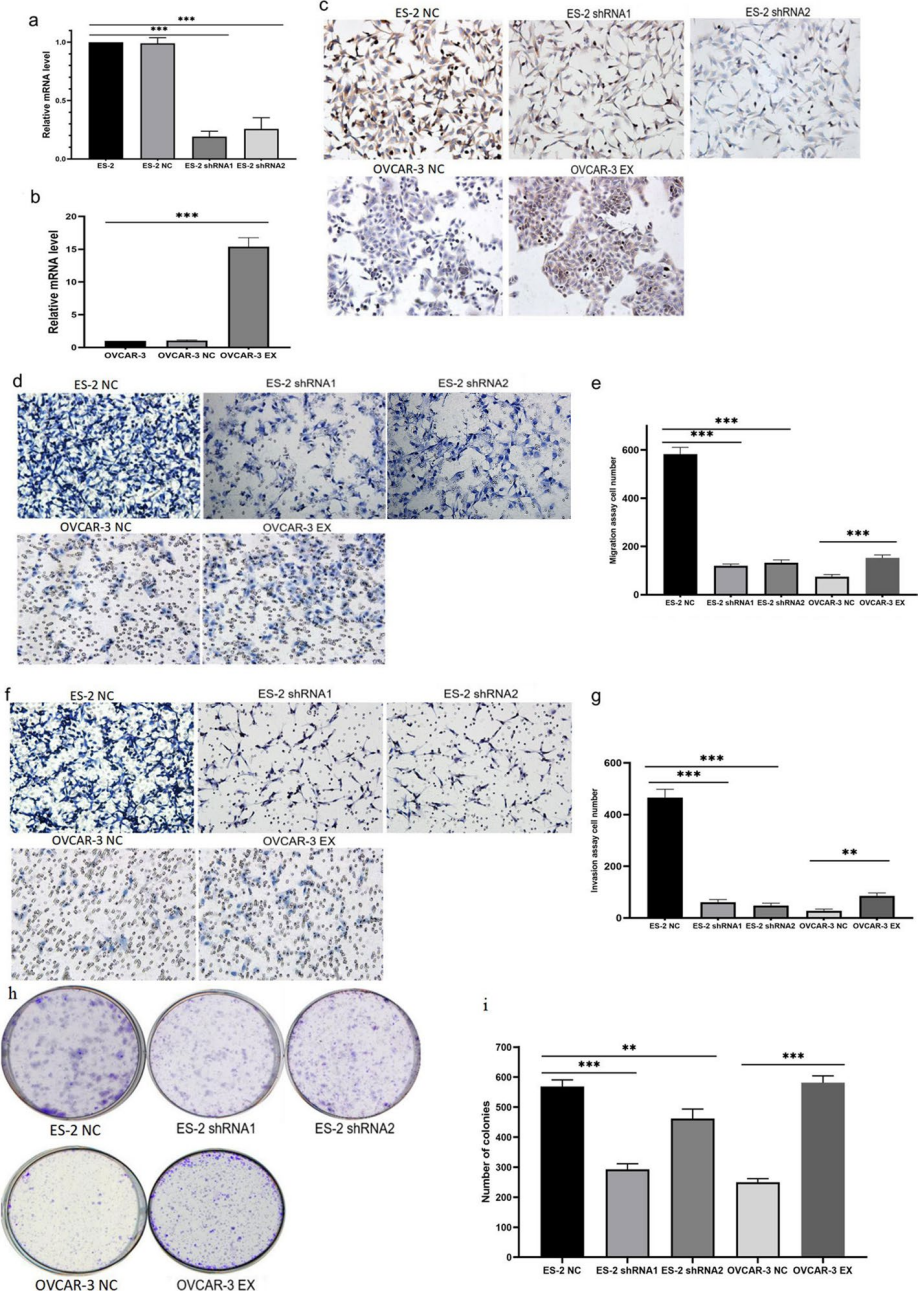
The verification of transfection efficiency and the changes in ovarian cancer cell migration, invasion and clonogenesis after transfection. RT-qPCR was used to verify EFEMP2 mRNA expression in (**a**) ES-2 cells after shRNA transfection, and (**b**) OVCAR-3 cells after over-expression transfection. **c** ICC verified EFEMP2 protein expression in ES-2, ES-2 shRNA1, ES-2 shRNA2, OVCAR-3, and OVCAR-3 EX cells. **d** The change in the migration ability of the cells after shRNA transfection and over-expression transfection, and (**e**) the histogram displayed the number of cells passing through the chamber membrane. **f** The change in the invasion ability of the cells after shRNA transfection and over-expression transfection, and (**g**) the histogram displayed the number of cells passing through the chamber membrane coated with Matrigel. **h** Plate clone assays were used to detect the clonogenic ability of ES-2, ES-2 shRNA1, ES-2 shRNA2, OVCAR-3, and OVCAR-3 EX cells, and **i** the histogram indicated the number of clones formed. Magnification × 200, *p < 0.05, **p < 0.01, or***P < 0.001

To further explore the regulatory function of EFEMP2 in ovarian cancer, ES-2 and OVCAR-3 cells with the highest and lowest EFEMP2 expression were selected for transfection experiments. Then shRNAs with different sequences were used to modulate the expression of EFEMP2 in ES-2 cells to create stable EFEMP2-silencing cell lines (ES-2 shRNA1, ES-2 shRNA2). In contrast, EFEMP2 expression in OVCAR-3 cells was increased to form a stable EFEMP2-overexpressing cell line (OVCAR-3 EX). The negative controls for transfection were ES-2 NC and OVCAR-3 NC. RT-qPCR and ICC results verified the transfection efficiency, and EFEMP2 expression in ES-2 shRNA1 and ES-2 shRNA2 was significantly reduced (Fig. 2a, c and Additional file 1: Table S4). In OVCAR-3 EX cells, the results were opposite (Fig. 2b, c and Additional file 1: Table S5), and the expression of EFEMP2 increased significantly.

### EFEMP2 can promote the migration, invasion and clonogenicity of ovarian cancer cells

The number of cells passing through the chamber membrane represented the ability to migrate or invade. After the knockdown of EFEMP2 expression, the number of ES-2 shRNA1 and ES-2 shRNA2 cells passing through the chamber membrane was lower than that of ES-2 NC cells, indicating that EFEMP2 silencing inhibited cell migration capacity, in contrast, EFEMP2 overexpression increased the number of cells passing through the chamber membrane (Fig. 2d, e and Additional file 1: Table S6). The results were consistent for the invasion assay: EFEMP2 down-expression inhibited cells' ability to penetrate Matrigel and the chamber membrane, while EFEMP2 overexpression promoted the increase of cell invasion ability (Fig. 2f, g and Additional file 1: Table S7). The plate clone formation assay could reflect the cloning potential and cell population dependence. According to the number and size of clones formed in the clone plates, cells with high expression of EFEMP2 had higher cell clonogenicity, while the group with low expression of EFEMP2 had the opposite effect (Fig. 2h, i and Additional file 1: Table S8). Taken together, reducing EFEMP2 expression could inhibit the migration, invasion and clonogenicity of ovarian cancer cells, whereas increasing EFEMP2 expression could promote the migration, invasion and clonogenicity of ovarian cancer cells.

### EFEMP2 can promote ovarian cancer cell EMT progression

**Fig. 3:**
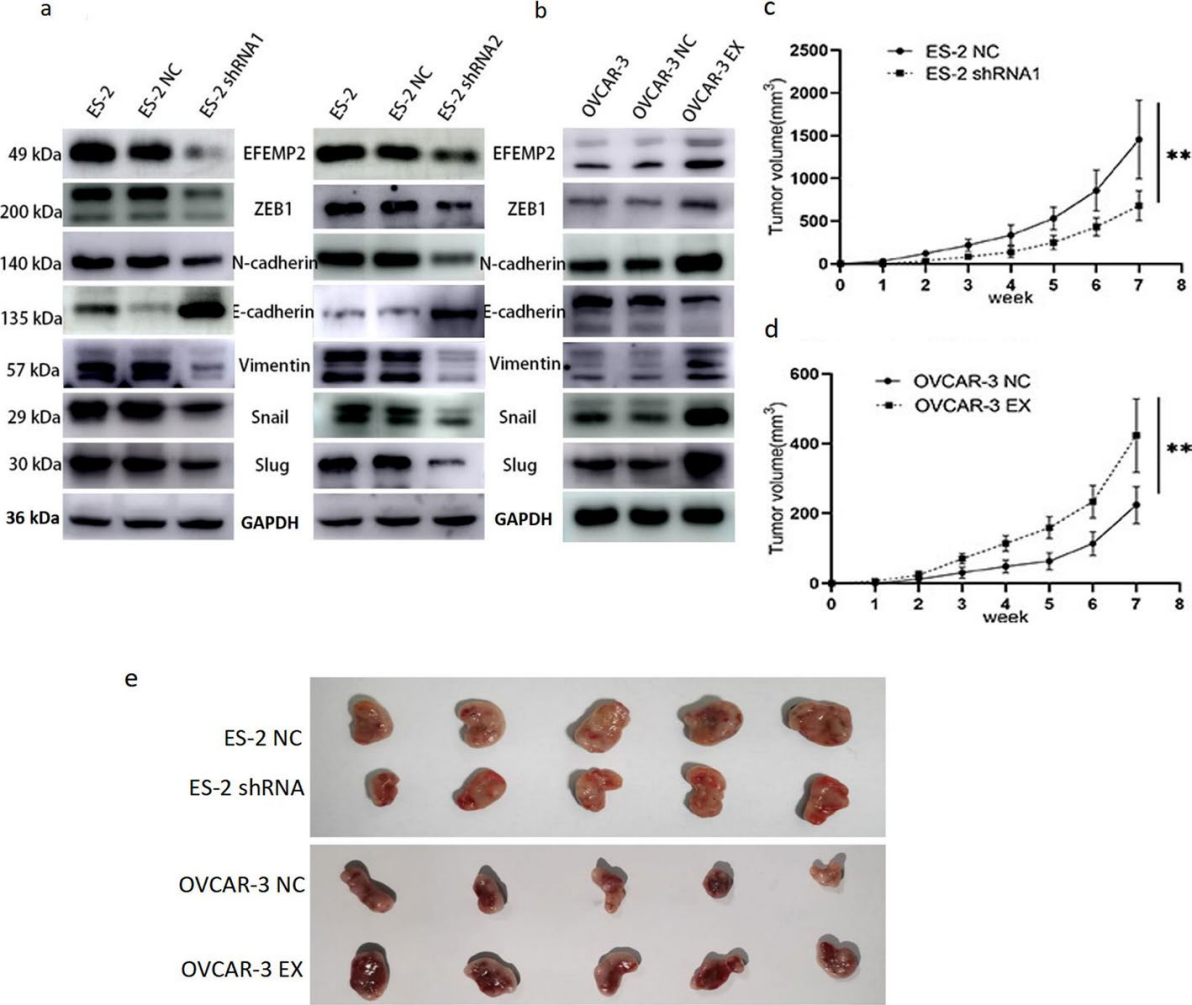
Effects of up-regulated or down-regulated EFEMP2 on EMT-related gene expression and in vivo growth of subcutaneous grafts. Western blot analysis was performed to detect (**a**) the effect of down-regulated EFEMP2 on the expression of EMT-related genes, and (**b**) the influence of EFEMP2 over-expression on the expression of EMT-related genes. **c** The graph illustrated the growth rate and size of tumors in nude mice after shRNA transfection. **d** The graph illustrated the growth rate and size of tumors in nude mice after over-expression transfection. **e** The images of subcutaneous graft tumors after inoculation with ES-2, ES-2 shRNA1, OVCAR-3, or OVCAR-3 EX cells. *p < 0.05, **p < 0.01, or***P < 0.001

EMT confers cancer cells an increased likelihood of metastasis [33]. E-cadherin expression in ES-2 shRNA1 and ES-2 shRNA2 cells was higher than that in ES-2 and ES-2 NC cells, in contrast, the expression of N-cadherin, Vimentin, Snail, Slug and Zeb1 was lower in EFEMP2-silencing cells than in ES-2 and ES-2 NC cells, as indicated by western blot (Fig. 3a). In OVCAR-3 EX cells, E-cadherin expression was lower, but other related genes expression was higher than that in OVCAR-3 and OVCAR-3 NC cells (Fig. 3b). Therefore, we believed that EFEMP2 could promote the EMT process of ovarian cancer cells.

### EFEMP2 can promote the growth of subcutaneous grafts in nude mice in vivo

Five nude mice were implanted in each cell line (ES-2 NC, ES-2 shRNA1, OVCAR-3 NC, and OVCAR-3 EX), and the tumor formation rate in all groups was 100%. As shown in Fig. 3c–e, Additional file 1: Tables S9 and S10, the tumor growth rate of EFEMP2-silencing group slowed down, and the mean tumor volume was significantly lower than that of control group. On the contrary, the tumor growth rate was significantly accelerated in the EFEMP2 overexpression group, and the tumor volume was greater than that in the control group. Therefore, high EFEMP2 expression could promote ovarian cancer cell proliferation in vivo.

### EFEMP2 can inhibit PD-L1 expression

**Fig. 4:**
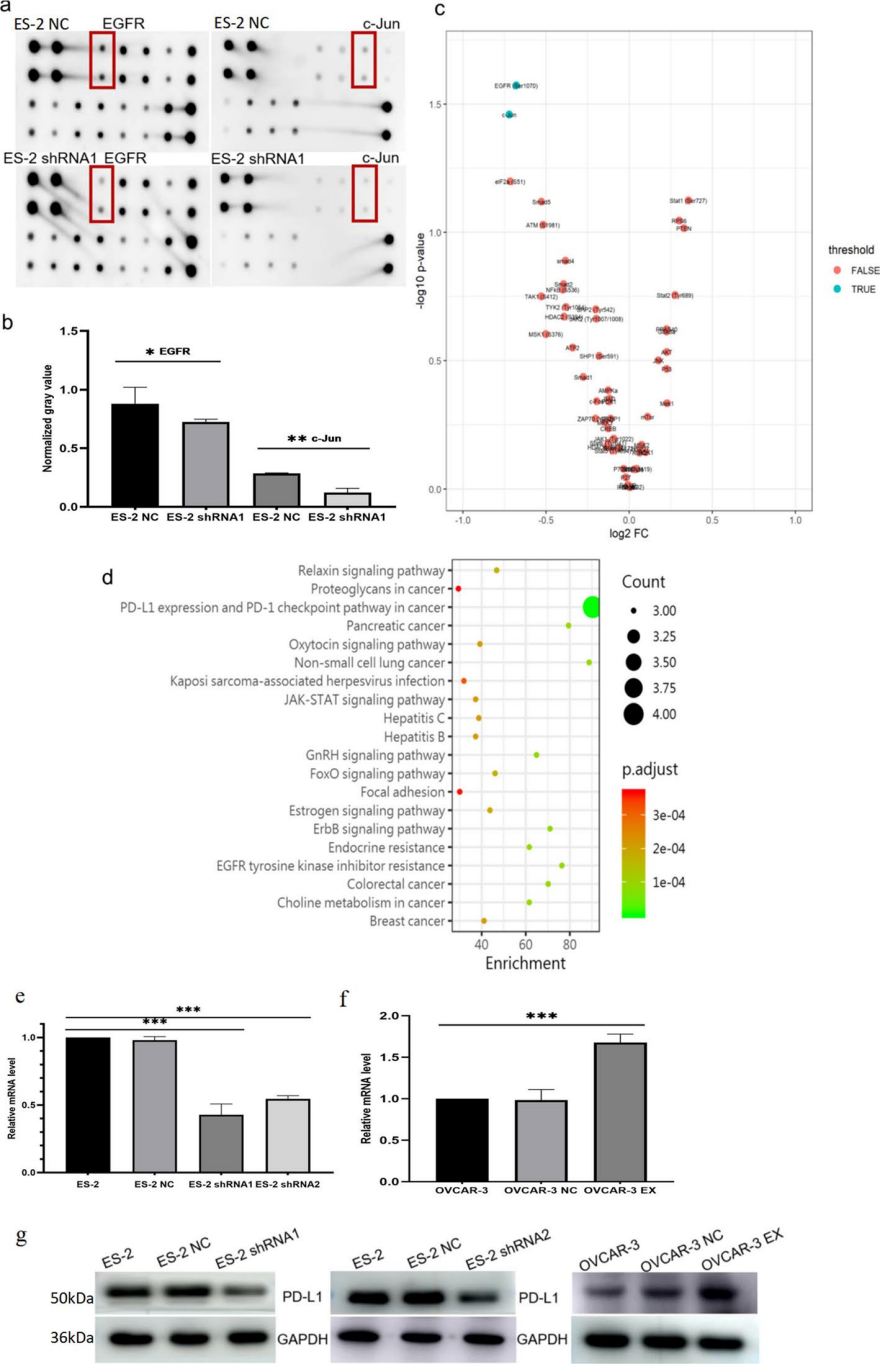
The downstream pathway of EFEMP2 was detected by phosphorylation pathway profiling array and the relationship between EFEMP2 and PD-L1 expression. ES-2 and ES-2 shRNA1 cell extracts were subjected to the phosphorylation pathway profiling array, (**a**) the membranes were scanned and (**b**) the histogram shows the normalization of target genes by positive control points. **c** Different EFEMP2 expression cells were used for proteomic analysis and the volcano map of differentially expressed genes was drawn. **d** A bubble chart showed the enrichment analysis of the KEGG pathway of differentially expressed genes. RT-qPCR was used to verify PD-L1 mRNA expression in **e** ES-2, ES-2 NC, ES-2 shRNA1, and ES-2 shRNA2 cells after EFEMP2 down-regulation, and (**f**) OVCAR-3, OVCAR-3 NC, and OVCAR-3 EX cells after EFEMP2 over-expression. **g** Western blot was used to detect the protein expression of PD-L1 in ES-2, ES-2 NC, ES-2 shRNA1, ES-2 shRNA2, OVCAR-3, OVCAR-3 NC, and OVCAR-3 EX cells, respectively. *p < 0.05, **p < 0.01, or***P < 0.001

To explore the significantly altered pathways in EFEMP2 knockdown cells, we performed a phosphorylation pathway profiling array on ES-2 NC and ES-2 shRNA1 cell extracts. As shown in Figs. 4a–c and Additional file 1: Table S11, epidermal growth factor receptor (EGFR) and c-Jun were the most significantly reduced signals. Then, KEGG pathway enrichment analysis was performed for differential genes, and it was found that PD-L1 expression and PD-L1 checkpoint pathway were significantly enriched in ovarian cancer (Fig. 4d). This suggested that the PD-L1 signaling pathway in EFEMP2 down-regulated cells might be repressed. Next, we conducted RT-qPCR (Fig. 4e, f, Additional file 1: Tables S12 and S13) and western blot (Fig. 4g) to examine the PD-L1 expression in ES-2, ES-2 NC, ES-2 shRNA1, ES-2 shRNA2, OVCAR-3, OVCAR-3 NC, and OVCAR-3 EX cells. The results revealed that the expression of PD-L1 was decreased in the EFEMP2-silencing group, but increased in the EFEMP2 overexpressing group. From the above results, the expression of EFEMP2 could directly affect the expression of PD-L1, and there was a positive correlation between them.

### EFEMP2 binds to EGFR to activate ERK1/2/c-Jun pathway to regulate PD-L1 expression

**Fig. 5:**
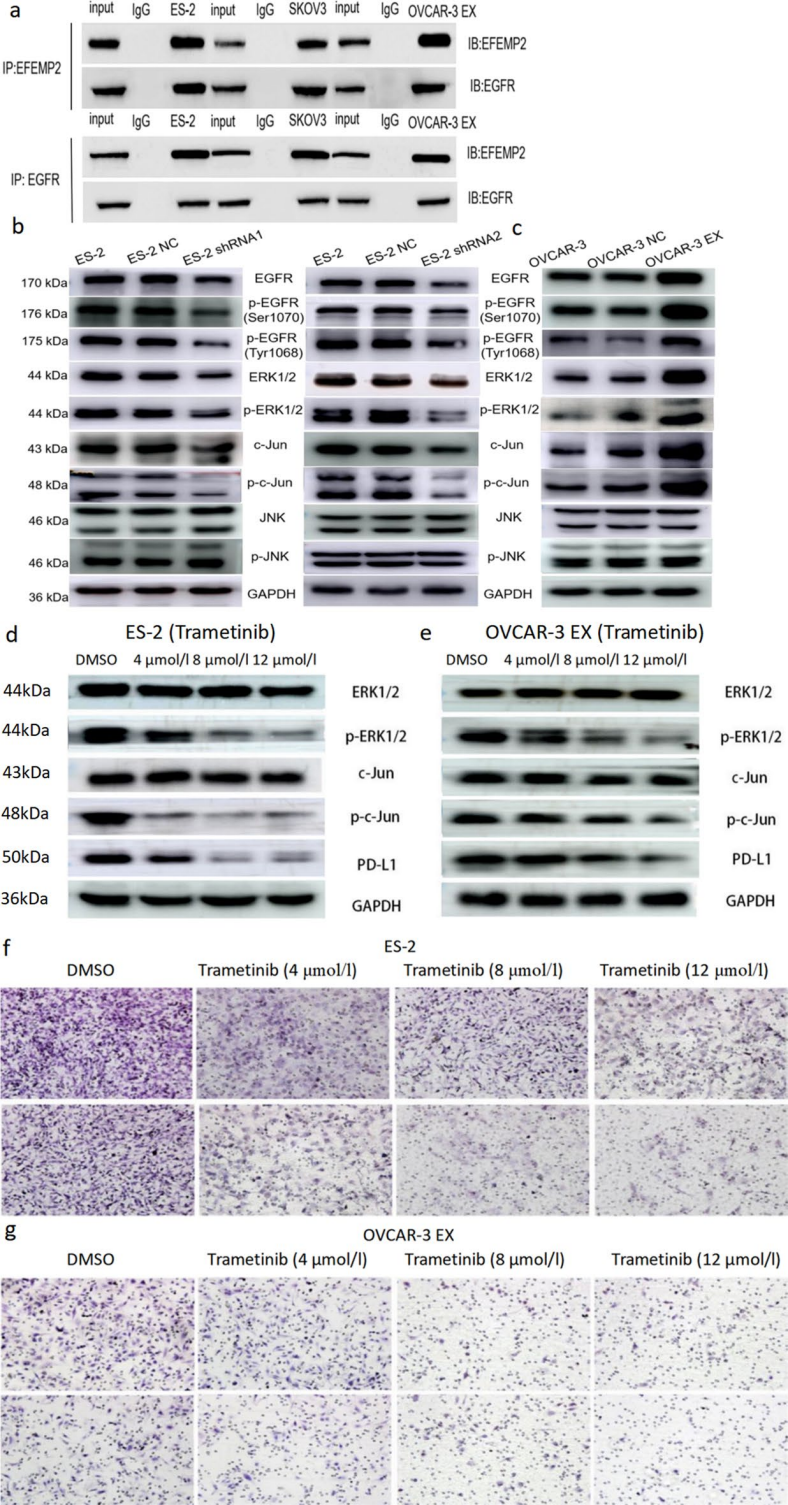
Confirmation of EFEMP2-EGFR binding and the effect of down or up expression of EFEMP2 on ERK1/2/c-Jun pathway or the JNK/c-Jun pathway. **a** By co-IP assay, the EGF-related protein EFEMP2 was confirmed to bind to EGFR. **b** By western blot, EFEMP2 knockdown significantly reduced the expression and phosphorylation level of EGFR, ERK1/2 and c-Jun, however, the expression and phosphorylation level of JNK remained unchanged. **c** Conversely, EFEMP2 overexpression increased EGFR, ERK1/2 and c-Jun expression and their phosphorylation levels, but had no effect on the expression and phosphorylation level of JNK. Western blot was used to analyze the expression and phosphorylation level of ERK1/2/c-Jun pathway and PD-L1 expression in (**d**) ES2 and (**e**) OVCAR-3 EX after trametinib (0 μmol/l, 4 μmol/l, 8 μmol/l, and 12 μmol/l) treatment. The transwell chamber was used to detect the migration and invasion ability of (**f**) ES-2 and (**g**) OVCAR-3 EX cells after trametinib (0 μmol/l, 4 μmol/l, 8 μmol/l, and 12 μmol/l) treatment. Magnification × 200

By co-IP, we confirmed that EGF-related protein EFEMP2 could bind EGFR (Fig. 5a), and the phosphorylation pathway profiling array analysis showed that the downstream pathway of EFEMP2 might be the c-Jun pathway, we further examined whether EFEMP2-EGFR binding could activate the ERK1/2/c-Jun pathway or the JNK/c-Jun pathway. By western blot (Fig. 5b, c), EFEMP2 knockdown significantly reduced EGFR expression and inhibited its phosphorylation levels at Ser1070 and Tyr1068, conversely, EFEMP2 overexpression increased EGFR expression and its phosphorylation level. At the same time, when EFEMP2 was down-regulated, the expression of ERK1/2 and c-Jun and their phosphorylation levels were also decreased, and when EFEMP2 was over-expressed, the situation was opposite. However, the expression and phosphorylation levels of JNK remained unchanged regardless of whether EFEMP2 was down-expressed or over-expressed. So we concluded that EFEMP2-EGFR binding could activate the ERK1/2/c-Jun pathway to regulate PD-L1 expression.

### Both Trametinib, a MEK inhibitor, and Afatinib, an EGFR inhibitor, can reduce the migration and invasion ability of ES-2 and OVCAR-3 EX cells and the expression of PD-L1

**Fig. 6:**
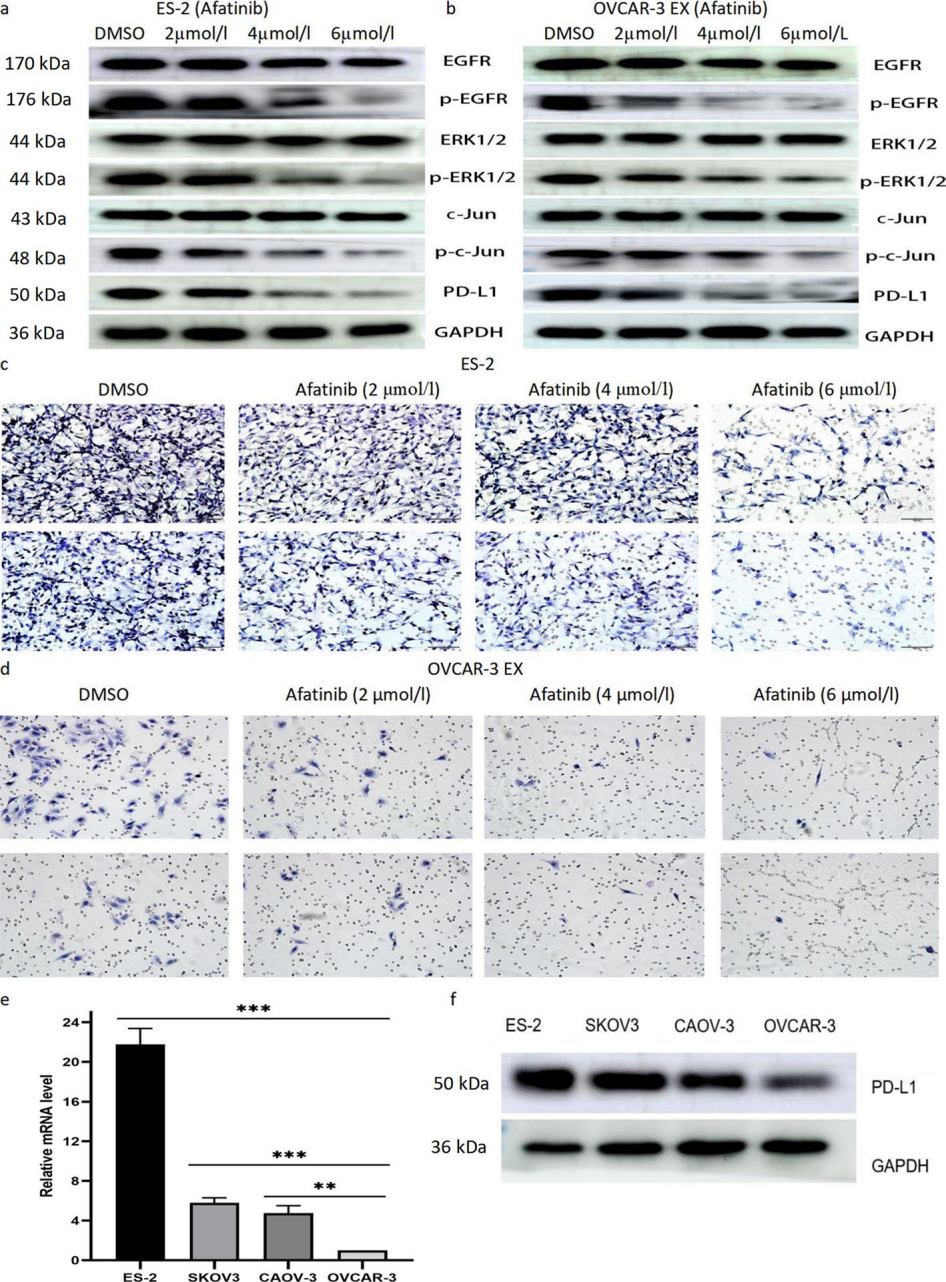
The effects of Afatinib on the EGFR/ERK1/2 /c-Jun pathway and the expression of PD-L1. Western blot was used to analyze the expression and phosphorylation level of EGFR/ERK1/2/c-Jun pathway and PD-L1 expression in (**a**) ES2 and (**b**) OVCAR-3 EX after Afatinib (0 μmol/l, 2 μmol/l, 4 μmol/l, and 6 μmol/l) treatment. The transwell chamber was used to detect the migration and invasion ability of (**c**) ES-2 and (**d**) OVCAR-3 EX cells after Afatinib (0 μmol/l, 2 μmol/l, 4 μmol/l, and 6 μmol/l) treatment. PD-L1 mRNA and protein levels in the four ovarian cancer cell lines were detected by RT-qPCR (**e**) and western blot (**f**). Magnification × 200. *p < 0.05, **p < 0.01, or***P < 0.001

To further clarify the effect of EFEMP2 on PD-L1 expression through EGFR/ERK1/2/c-Jun signaling pathway, we used MEK inhibitor Trametinib and EGFR inhibitor Afatinib on ES-2 and OVCAR-3 EX cells, respectively. Then, the changes of PD-L1 expression and cell migration and invasion ability before and after inhibitor treatment were analyzed. In a dose-dependent manner, Trametinib hampered the expression of PD-L1 by inhibiting the phosphorylation of ERK1/2 and c-Jun in ES-2 (Fig. 5d) and OVCAR-3 EX cells (Fig. 5e), and obviously inhibited the migrative and invasive ability of ES-2 (Fig. 5f) and OVCAR-3 EX cells (Fig. 5g) with high EFEMP2 expression. Afatinib weakened the phosphorylation levels of EGFR, ERK1/2 and c-Jun, thereby reducing the expression level of PD-L1 in ES-2 (Fig. 6a) and OVCAR-3 EX cells (Fig. 6b). Meanwhile, Afatinib could significantly inhibit the migrative and invasive ability of ES-2 (Fig. 6c) and OVCAR-3 EX cells (Fig. 6d), and the inhibitory effect was enhanced with the increase of dose.

### Acquisition of PD-L1 stable down- and over-expression cell models

**Fig. 7:**
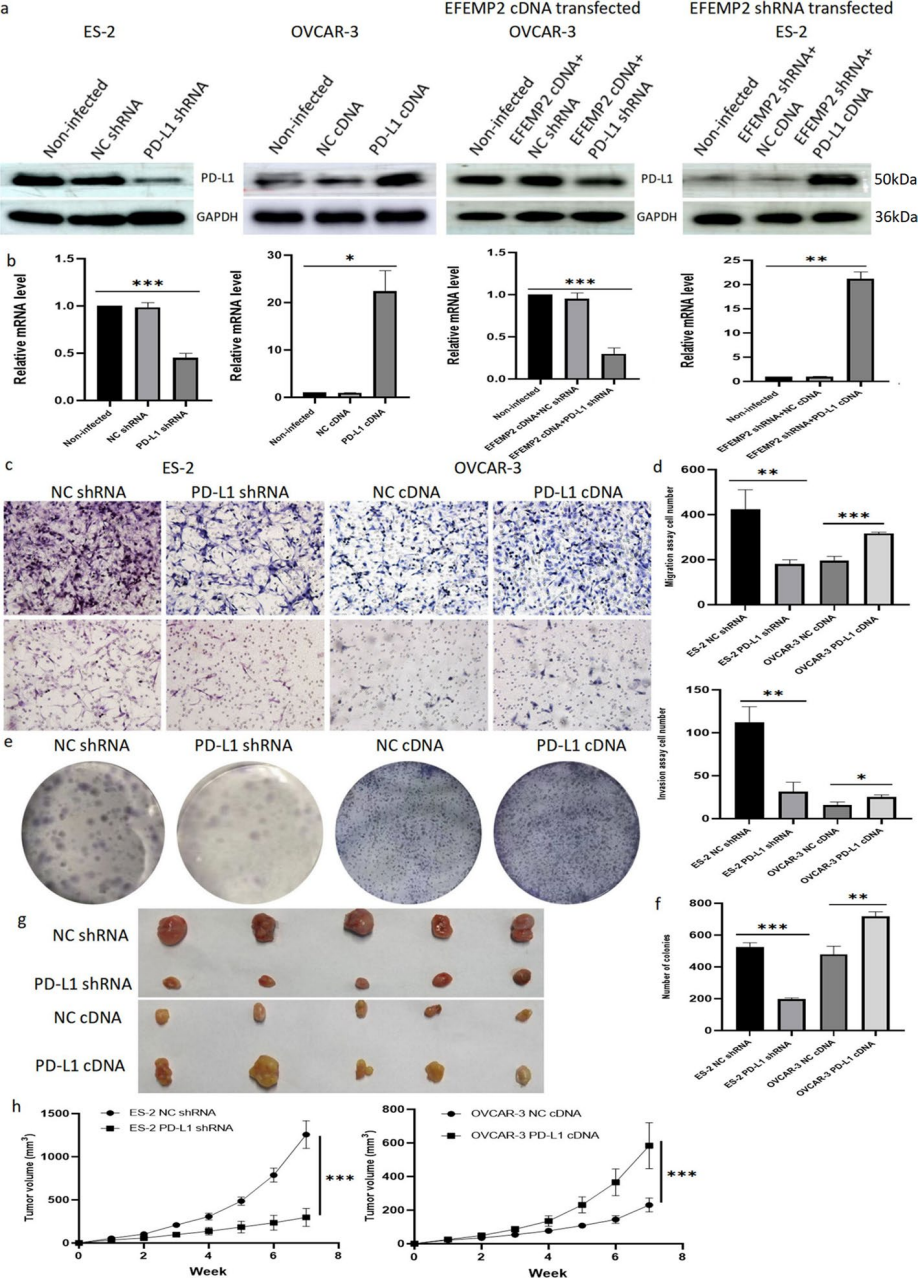
Acquisition of PD-L1 stable down- and over-expression cell models and the effects of PD-L1 up- or down- regulation on the migration, invasion and clonogenicity of ovarian cancer cells in vitro and the growth of subcutaneous graft tumor in vivo. Western blot (**a**) and RT-qPCR (**b**) verified the transfection effects, and PD-L1 expression was significantly decreased in PD-L1 shRNA transfected group and EFEMP2 cDNA + PD-L1 shRNA transfected group, meanwhile the expression of PD-L1 was remarkably increased in PD-L1 cDNA transfected group and EFEMP2 shRNA + PD-L1 cDNA transfected group. **c** Changes in cell migration and invasion ability after transfection with PD-L1 shRNA or cDNA, and (**d**) the histogram displayed the number of cells passing through the chamber membrane in migration and invasion assay. **e** Plate clone assays were used to detect the change of cloning ability before and after transfection of PD-L1 shRNA or cDNA, and (**f**) the histogram indicated the number of clones formed in the down-expression group and overexpression group. **g** The images of subcutaneous graft tumors after inoculation in PD-L1 down-expression group and PD-L1 overexpression group. **h** The graph illustrated the growth rate and size of tumors in nude mice after PD-L1 shRNA or cDNA transfection. Magnification × 200. *p < 0.05, **p < 0.01, or ***p < 0.001

The expression of PD-L1 in four types of ovarian cancer cells with different invasive abilities was detected by RT-qPCR (Fig. 6e and Additional file 1: Table S14) and western blot (Fig. 6f). The results showed that the expression of PD-L1 increased with the enhancement of cell invasion ability, and the highest expression was found in ES-2 cells with high invasive ability. Next, OVCAR-3 cells and EFEMP2 shRNA transfected ES-2 cells with low PD-L1 expression were selected to construct PD-L1 overexpression cell models, which were expressed as PD-L1 cDNA transfected group and EFEMP2 shRNA + PD-L1 cDNA transfected group, respectively. Meanwhile, ES-2 cells and EFEMP2 cDNA transfected OVCAR-3 cells with high PD-L1 expression were chosen to build PD-L1 low expression cell models, which were labeled as PD-L1 shRNA transfected group and EFEMP2 cDNA + PD-L1 shRNA transfected group. Western blot (Fig. 7a) and RT-qPCR (Fig. 7b and Additional file 1: Table S15) verified the transfection effects, and the expression of PD-L1 was significantly decreased in the down-expression group, while remarkably increased in the overexpression group.

### PD-L1 also has a clear capacity to promote the migration, invasion and clonogenicity of ovarian cancer cells in vitro and the growth of subcutaneous graft tumor in vivo

By transwell chambers, the invasion and migration ability of cells with down-expression of PD-L1 was decreased, while overexpression of PD-L1 enhanced cell invasion and migration (Fig. 7c, d and Additional file 1: Table S16). At the same time, PD-L1 knockdown restrained the number and volume of clones formed in the plate, but the overexpression of PD-L1 had the opposite effect (Fig. 7e, f and Additional file 1: Table S17). We also observed that in xenografted tumor models, low PD-L1 expression retarded tumor growth, and overexpression of PD-L1 accelerated it (Fig. 7g, h and Additional file 1: Table S18).

### PD-L1 is required for EFEMP2 to promote ovarian cancer cells proliferation and invasion in vitro and in vivo

**Fig. 8:**
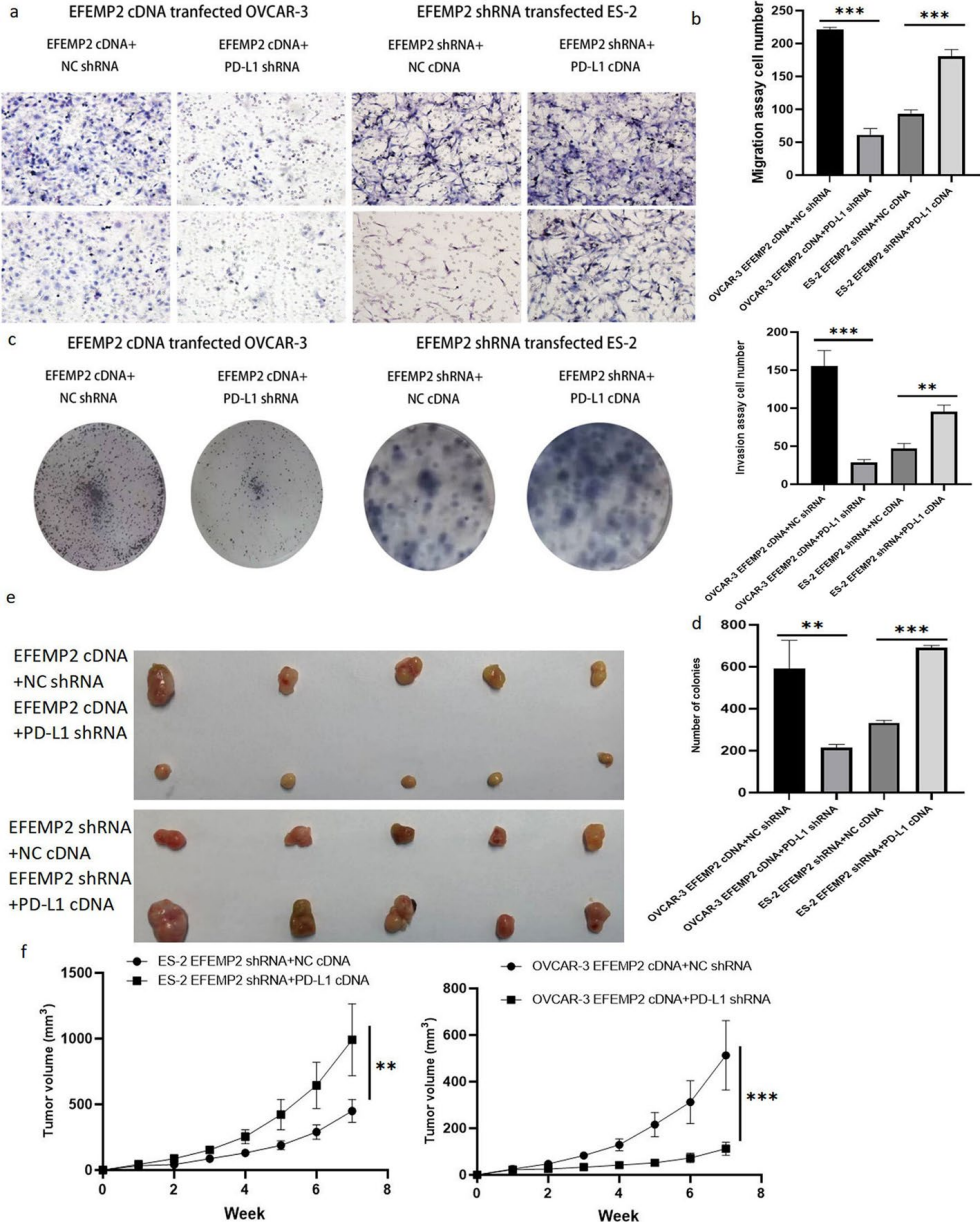
The important role of PD-L1 in EFEMP2 promoting the invasion of ovarian cancer cells. **a** After transwell assay, in OVCAR-3 cells co-transfected with EFEMP2 cDNA and PD-L1 shRNA, the down-regulation of PD-L1 significantly reversed the increase in invasion and mobility caused by overexpression of EFEMP2, conversely, overexpression of PD-L1 in ES-2 cells co-transfected with EFEMP2 shRNA and PD-L1 cDNA enhanced the invasion and mobility inhibited by EFEMP2 knockdown. **b** the histogram displayed the number of cells passing through the chamber membrane in migration and invasion assay. **c** By plate cloning formation assay, the down-regulation of PD-L1 significantly inhibited the increase of cell cloning induced by EFEMP2 overexpression, on the contrary, overexpression of PD-L1 significantly reversed the reduction in the number of cell clones caused by EFEMP2 knockdown. **d** the histogram indicated the number of clones formed. **e** The images of subcutaneous graft tumors after inoculation in the PD-L1 down-regulated and up-regulated groups. **f** The graph illustrated the growth rate and size of tumors in nude mice after PD-L1 shRNA or cDNA transfection. Magnification × 200. *p < 0.05, **p < 0.01, or ***p < 0.001

Further experiments were conducted to verify whether PD-L1 played a decisive role in the EFEMP2-induced enhancement in the proliferation and invasion of ovarian cancer cells. EFEMP2 cDNA and PD-L1 shRNA were co-transfected into OVCAR-3 cells to observe whether PD-L1 silencing would block the promotion effect of EFEMP2 overexpression on the proliferation and invasion of ovarian cancer cells. Similarly, by co-transfecting EFEMP2 shRNA and PD-L1 cDNA into ES-2 cells, we investigated whether overexpression of PD-L1 could reverse the blocking effect of EFEMP2 knockout on the proliferation and invasion of ovarian cancer cells. After transwell assay, in OVCAR-3 cells co-transfected with EFEMP2 cDNA and PD-L1 shRNA, the down-regulation of PD-L1 significantly reversed the increase in invasion and mobility caused by overexpression of EFEMP2, conversely, overexpression of PD-L1 in ES-2 cells co-transfected with EFEMP2 shRNA and PD-L1 cDNA enhanced the invasion and mobility inhibited by EFEMP2 knockdown (Fig. 8a, b and Additional file 1: Table S19). By plate cloning formation assay, the down-regulation of PD-L1 significantly inhibited the increase of cell cloning induced by EFEMP2 overexpression, on the contrary, overexpression of PD-L1 significantly reversed the reduction in the number of cell clones caused by EFEMP2 knockdown (Fig. 8c, d and Additional file 1: Table S20). The results of subcutaneous tumor grafts in nude mice showed that knockout of PD-L1 significantly inhibited the promotion effect of EFEMP2 overexpression on the growth of subcutaneous transplanted tumors; however, overexpression of PD-L1 restored the growth of subcutaneous transplanted tumors inhibited by EFEMP2 knockout (Fig. 8e, f and Additional file 1: Table S21). In one word, PD-L1 was crucial for EFEMP2 to promote ovarian cancer cells invasion and migration in vitro and in vivo.

### PD-L1 is extremely important in the process of EFEMP2 promoting EMT progression in ovarian cancer cells

**Fig. 9:**
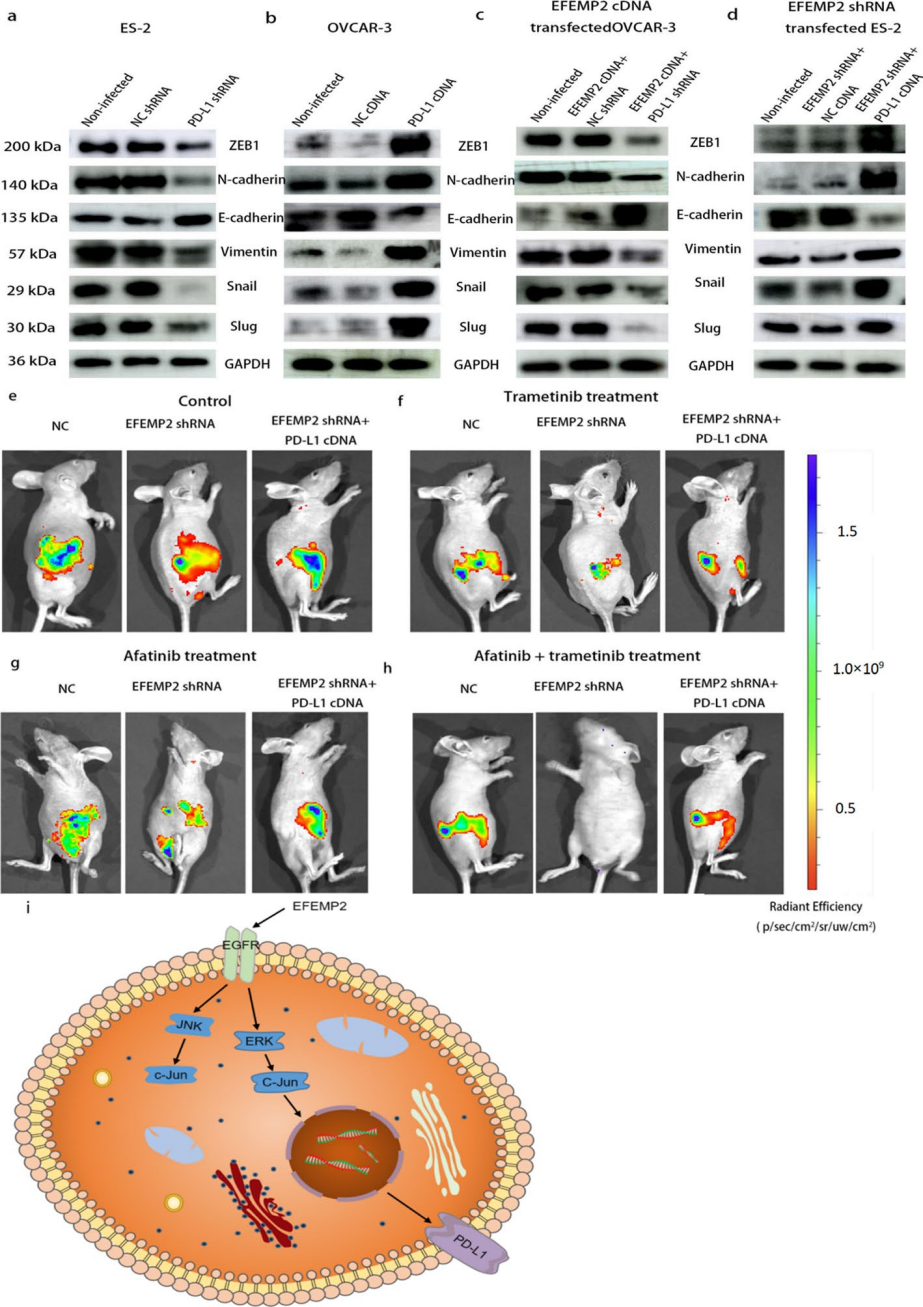
The important role of PD-L1 in EFEMP2 promoting EMT progression and the inhibitory effect of EFEMP2 knockdown on the abdominal dissemination of ovarian cancer cells in nude mice. **a** PD-L1 knockout significantly increased the levels of E-cadherin, while down-regulated the levels of N-cadherin, vimentin, Slug, Snail and Zeb1, indicating that downregulation of PD-L1 inhibited EMT process. **b** Up-regulation of PD-L1 induced EMT, accompanied by decreased E-cadherin levels, and increased N-cadherin, vimentin, Slug, Snail and Zeb1 levels. **c** Downregulated PD-L1 significantly inhibited the EMT process induced by EFEMP2 overexpression, accompanied by an increase in E-cadherin level, and decreased levels of N-cadherin, vimentin, Slug, Snail and Zeb1. **d** However, overexpressed PD-L1 restored the EMT process that had been hampered by EFEMP2 knockout. **e** EFEMP2 down-expression could significantly inhibit the spread of ovarian cancer cells in the abdominal cavity of nude mice, but the overexpression of PD-L1 drastically promoted the process. **f** The therapeutic effect of Trametinib in inhibiting abdominal spread of ES-2 NC, EFEMP2 shRNA infected cells and EFEMP2 shRNA and PD-L1 cDNA co-transfected cells. **g** The therapeutic effect of Afatinib in inhibiting abdominal spread of ES-2 NC, EFEMP2 shRNA infected cells and EFEMP2 shRNA and PD-L1 cDNA co-transfected cells. **h** Effect of Trametinib and Afatinib combination therapy in inhibiting abdominal spread of ovarian cancer cells. **i** EFEMP2 could induce PD-L1 regulation in ovarian cancer cells by activating the EGFR/ERK1/2/c-Jun pathway rather than the JNK/c-Jun pathway

Consistent with EFEMP2, PD-L1 could also promote EMT progression in ovarian cancer cells. PD-L1 knockout significantly increased the levels of epithelial markers E-cadherin, while down-regulated the levels of interstitial markers N-cadherin and vimentin, in addition, the expressions of Slug, Snail and Zeb1 transcription factors were also significantly decreased, indicating that downregulation of PD-L1 inhibited EMT process (Fig. 9a). Up-regulation of PD-L1 induced EMT, decreased E-cadherin levels, and increased N-cadherin, vimentin, Slug, Snail and Zeb1 levels (Fig. 9b). In EFEMP2 cDNA and PD-L1 shRNA co-transfected OVCAR-3 cells and EFEMP2 shRNA and PD-L1 cDNA co-transfected ES-2 cells, downregulated PD-L1 significantly inhibited the EMT process induced by EFEMP2 overexpression, accompanied by an increase in E-cadherin level, and decreased levels of N-cadherin, vimentin, Slug, Snail and Zeb1 (Fig. 9c). However, overexpressed PD-L1 restored the EMT process that had been hampered by EFEMP2 knockout (Fig. 9d).

### EFEMP2 knockdown can significantly inhibit the abdominal dissemination of ovarian cancer cells in nude mice, and the overexpression of PD-L1 reverses this inhibitory effect

ES-2 NC, EFEMP2 shRNA infected cells and EFEMP2 shRNA and PD-L1 cDNA co-transfected cells were injected intraperitoneally in nude mice, with 1.5 × 10^7^ cells for each mouse to establish an ascites tumor model. Ten days after the models were built, Afatinib, Trametinib, and Afatinib combined with Trametinib were injected intraperitoneally for 7 consecutive days. The results showed that EFEMP2 down-expression could significantly inhibit the spread of ovarian cancer cells in the abdominal cavity of nude mice, but the overexpression of PD-L1 drastically promoted the process (Fig. 9e). Trametinib treatment group (Fig. 9f), afatinib treatment group (Fig. 9g), and Afatinib and trametinib combined treatment group (Fig. 9h) could inhibit the formation of intraperitoneal spread of the three groups of cells, and the combination treatment group has the most obvious effect. Moreover, the effect of the three treatments on EFEMP2 down-expression group was particularly significant, but the effect on PD-L1 overexpression group was poor. In conclusion, EFEMP2 promoted the invasion and dissemination of ovarian cancer cells in vivo, and PD-L1 was essential in this process.

## Discussion

Our team previously demonstrated that EFEMP2 was associated with tumor progression of ovarian cancer and poor prognosis [13]. Our subsequent studies at least partially revealed the mechanism of EFEMP2 in the invasion and metastasis of ovarian cancer. We proved for the first time that EFEMP2 could bind to EGFR to activate ERK1/2/c-Jun pathway and regulate PD-L1 expression (Fig. 9I), furthermore, PD-L1 was extremely important for EFEMP2 to promote ovarian cancer cells invasion in vitro and in vivo.

It was previously reported that EFEMP2 was an extracellular glycoprotein, which played a key role in tumors [34], and it was considered to be a candidate oncogene product [7]. Our study found that high expression of EFEMP2 was associated with low differentiation of ovarian cancer, advanced clinical stage, and positive lymph node metastasis, but not with the tissue type of ovarian cancer (including serous cystadenocarcinoma, mucinous cystadenocarcinoma, and endometrioid carcinoma), and that EFEMP2 was highly expressed in ovarian cancer cells with high migration and invasion capacity [13]. PD-L1 was also highly expressed in ovarian cancer tissues, and there was no statistically significant difference in expression among different tissue types, including serous carcinoma, endometrioid carcinoma, mucinous carcinoma, granular cell carcinoma and clear cell carcinoma [35]. Another study showed that PD-L1 was closely related to high malignancy, low chemotherapy response and poor prognosis of ovarian clear cell carcinoma [36]. The decreased expression of PD-L1 inhibits the invasion and migration of ovarian cancer cells [37].

In the same way, when EFEMP2 was knock-down, the migration and invasion abilities were also reduced, whereas the results were opposite in ovarian cancer cells overexpressing EFEMP2. The ability of epithelial cells to migrate and invade was through the EMT process [38–40]. In the process of EMT, a shift of cells toward the mesenchymal state changed the adhesion molecules expressed by the cells, causing them to adopt migration and invasion behaviors, inducing cell proliferation and sowing new tumors [41, 42]. Knockdown of EFEMP2 could upregulate the epithelial marker E-cadherin, and downregulate the expression of N-cadherin, snail, vimentin, slug and Zeb1. However, overexpression of EFEMP2 could downregulate epithelial markers and upregulate mesenchymal markers. These results showed that in ovarian cancer cells EFEMP2-silencing could reduce EMT, otherwise, overexpression of EFEMP2 could promote EMT. The same results were also observed in osteosarcoma [15], but the opposite results in lung, endometrial, and bladder cancers [9, 10, 12]. This might be because of the inherent heterogeneity of tumor cells and the tumor microenvironment [41]. We further conducted tumor formation and dissemination models in nude mice. The tumorigenicity of cancer cells transfected with EFEMP2 shRNA was worse, the tumor growth rate was slower, and the average tumor volume was smaller, moreover, tumor abdominal dissemination was suppressed. Cancer cells that overexpressed EFEMP2 were more prone to tumorigenesis. Although this further verified the associated relationship between EFEMP2 expression and ovarian cancer, the current mechanism by which EFEMP2 affects ovarian tumors warrants further research.

Based on the phosphorylation pathway profiling array, EGFR and c-Jun were both lowly expressed after EFEMP2 was knocked down in ovarian cancer cells. Then the differential genes were analyzed by KEGG pathway enrichment analysis, and the PD-L1 expression and PD-L1 checkpoint pathway in cancer were significantly enriched. Recent studies have shown that in epithelial tumors, PD-L1 could affect a variety of intrinsic mechanisms of cancer cells, such as migration, invasion, proliferation, and apoptosis [43]. In ovarian cancer, PD-L1 expression was strongly associated with poor prognosis [44], and Anti-PD-L1/PD-1 immunotherapy for ovarian cancer has attracted increasing attention [45–47]. Some clinical efficacy has been observed in ovarian cancer, however, most patients initially did not respond to treatment [48]. Checkpoint inhibitors have limited effectiveness in ovarian cancer [49]. Therefore, further understanding of the mechanism of PD-L1 expression may help guide the development of immunotherapy for ovarian cancer. In our study, the upstream regulatory gene EFEMP2 of PD-L1 was found, and EFEMP2 could combine with EGFR to activate the EGFR/ERK1/2/c-Jun signaling pathway to at least partially regulate the expression of PD-L1. Similar to EFEMP2, PD-L1 was also highly expressed in aggressive cells. After EFEMP2 knockdown, PD-L1 also showed a downward trend. The decreased expression of PD-L1 could block the promotion effect of EFEMP2 overexpression on the invasion and migration of ovarian cancer cells, as well as the induction effect on EMT process. In vivo experiments in nude mice also demonstrated that PD-L1 overexpression could reverse the inhibitory effect of EFEMP2 down-expression on subcutaneous tumor growth and abdominal spread. It can be concluded that PD-L1 expression was associated with the expression of EFEMP2, and PD-L1 upregulation was partly caused by EFEMP2 activation and further influenced the progression of ovarian cancer.

The phosphorylation pathway profiling array showed that EGFR and c-Jun were the most significantly changed signals. EGFR was a driver of many tumorigeneses [50], and a target of many cancer treatments currently used in clinical practice [51]. The activation of EGFR controlled many cellular activities, such as apoptosis, proliferation, and growth [52]. EGFR was heavily involved in the EMT processes, which could induce EMT in a variety of tumorigenesis contexts [53, 54]. Experiments with the EGFR inhibitor AG1478 showed that EGF might promote the progression of ovarian cancer by activating the EGFR-ERK signaling pathway [55]. EFEMP1 was a secreted matricellular glycoprotein with five EGF-like domains and was considered to be a ligand of EGFR [56]. EFEMP1 and EFEMP2 had very similar sequences and domain structures, and they belonged to paralogs [34]. Co-IP experiments were conducted to analyze the interaction between EFEMP2 and EGFR, and as expected, they could combine with each other. Here, in ovarian cancer, our experiments strongly supported the notion that the oncogene EFEMP2 regulated the expression of PD-L1 driven by EGFR. To explore the molecular mechanism of EGFR activation and subsequent upregulation of PD-L1, we investigated the downstream pathways of EGFR. According to pathway profiling array, besides EGFR, c-Jun was another gene with remarkable change. Studies have shown that in ovarian cancer, the activation of transcription factor c-Jun was a necessary condition for the upregulation of PD-L1 [46]. The EGFR/ERK/c-Jun pathway could regulate PD-L1 expression in renal carcinoma [57]. The expression of PD-L1 in cancer cells could also be induced in a JNK/c-Jun-dependent manner [58, 59]. Therefore, we speculated that EGFR activation-caused upregulation of PD-L1 might be related to the activation of ERK1/2/c-Jun and JNK/ c-Jun pathways. Knockdown of EFEMP2 significantly decreased PD-L1 expression, and the expressions and their phosphorylation levels of EGFR/ERK1/2/c-Jun also showed a downward trend. However, these findings were reversed when EFEMP2 was overexpressed. Whereas, knockdown or overexpression of EFEMP2 had no effect on the JNK pathway. All these results showed that EGFR/ERK1/2/c-Jun pathway rather than the JNK/c-Jun pathway played an important role in EFEMP2-caused PD-L1 expression regulation. Another study showed that EFEMP2 was highly expressed in highly invasive cervical cancer cells Ca-Ski, and the up-regulation of EFEMP2 expression could further promote the proliferation and invasion of cervical cancer cells by inducing EMT through activation of Raf/MEK/ERK pathway [60]. It can be seen here that EFEMP2 is closely related to the ERK pathway.

To further verify the involvement of EGFR/ERK1/2/c-Jun pathway in EFEMP2 regulation of PD-L1, EGFR inhibitor Afatinib or MEK inhibitor Trametinib was used to block the EGFR or ERK1/2 activation in ES-2 and OVCAR-3 EX cells with high EFEMP2 expression. Specifically, by inhibiting the EGFR or ERK1/2 activation, the expression of PD-L1 was depressed and the migration and invasion of cells were decreased in a clear dose-dependent manner. Afatinib or Trametinib inhibited tumor intraperitoneal diffusion in ascites tumor models, and the combined treatment effect was more significant. Afatinib was approved by the FDA (Food and Drug Administration) in 2013 for the treatment of patients with metastatic NSCLC (non-small-cell lung cancer) and in 2016 for the treatment of squamous cell lung cancer [61]. But almost all patients will eventually develop disease progression due to the presence of acquired resistance to tyrosine kinase inhibitors [62]. The effect of afatinib alone was not obvious, and more and more studies were focusing on multi-drug combinations [63]. The combination of Afatinib and Trametinib to inhibit the ErbB family and MEK/ERK kinase may be a potentially effective treatment strategy for NSCLC [64]. Furthermore, in the treatment of oral squamous cell carcinoma, the combination of Afatinib and Trametinib had a synergistic antitumor effect in the mouse tumor-bearing model [65]. In this study, the combination of afatinib and trametinib was more effective than alone and had a more significant inhibitory effect on the intraperitoneal spread of ovarian cancer cells in vivo. Sufficient obstruction of PD-L1 expression by EGFR inhibitor Afatinib or MEK inhibitor Trametinib clearly indicated that EFEMP2's regulation of PD-L1 was accomplished through the EGFR/ERK1/2/c-Jun pathway. Targeted therapy against the source gene EFEMP2 is our future research direction, which may better inhibit the invasion and metastasis of ovarian cancer cells.

In summary, this study was the first to show that EFEMP2 was positively correlated with the invasion ability of ovarian cancer cells, its down-regulation inhibited the migrative, invasive and cloning capacity of cancer cells in vitro and suppressed the tumor proliferation and intraperitoneal diffusion in vivo, while its up-regulation did the opposite. Moreover, EFEMP2 could bind to EGFR to induce PD-L1 regulation in ovarian cancer, which was caused by the activation of EGFR/ERK1/2/c-Jun signaling. Based on the characteristics of EFEMP2 in our research on ovarian cancer, we believed that EFEMP2 might be used as a targeted drug to effectively inhibit the invasion and metastasis of ovarian cancer in the future.

## Supplementary Information

**Additional file 1: Table S1**. The number of cells passing through the chamber membrane in migration assay. **Table S2**. The number of cells passing through the chamber membrane in invasion assay. **Table S3**. The mRNA levels of EFEMP2 in four ovarian cancer cell lines. **Table S4**. The mRNA levels of EFEMP2 in ES-2 cells after shRNA transfection. **Table S5**. The mRNA levels of EFEMP2 in OVCAR-3 cells after overexpression transfection. **Table S6**. The number of cells passing through the chamber membrane in migration assay. **Table S7**. The number of cells passing through the chamber membrane in invasion assay. **Table S8**. The number of clones formed in the downexpression group and overexpression group. **Table S9**. The growth size of tumors in nude mice after shRNA transfection. **Table S10**. The growth size of tumors in nude mice after overexpression transfection. **Table S11**. The normalized gray value of target genes by positive control points. **Table S12**. The mRNA levels of PD-L1 in ES-2 cells after shRNA transfection. **Table S13**. The mRNA levels of PD-L1 in OVCAR-3 cells after overexpression transfection. **Table S14**. The mRNA levels of PD-L1 in four ovarian cancer cell lines. **Table S15**. The mRNA levels of PD-L1 in cells. **Table S16**. The number of cells passing through the chamber membrane in migration or invasion assay. **Table S17**. The number of clones formed in the downexpression group and overexpression group. **Table S18**. The growth size of tumors in nude mice after PD-L1 shRNA or cDNA transfection. **Table S19**. The number of cells passing through the chamber membrane in migration or invasion assay. **Table S20**. The number of clones formed in the downexpression group and overexpression group. **Table S21**. The growth size of tumors in nude mice after PD-L1 cDNA transfection

## Data Availability

All data generated or analyzed during this study are presented in the manuscript as far as possible.

## Abbreviations

EFEMP2: Fibulin-like extracellular matrix protein 2
PD-L1: Programmed death-1
EGF: Epidermal growth factor
PD-1: Programmed cell death 1
FBS: Fetal bovine serum
ICC: Immunocytochemistry
EMT: Epithelial–mesenchymal transition
co-IP: Co-immunoprecipitation
rpm: Revolutions per minute
