# Supplementary material for: EFEMP2 upregulates PD-L1 expression via EGFR/ERK1/2/c-Jun signaling to promote the invasion of ovarian cancer cells
Source: Cell Mol Biol Lett. 2023 Jul 7;28:53. doi: 10.1186/s11658-023-00471-8 (PMC10327401; doi:10.1186/s11658-023-00471-8)
Supplement: Supplementary file 1 — Additional file 1: Table S1. The number of cells passing through the chamber membrane in migration assay. Table S2. The number of cells passing through the chamber membrane in invasion assay. Table S3. The mRNA levels of EFEMP2 in four ovarian cancer cell lines. Table S4. The mRNA levels of EFEMP2 in ES-2 cells after shRNA transfection. Table S5. The mRNA levels of EFEMP2 in OVCAR-3 cells after overexpression transfection. Table S6. The number of cells passing through the chamber membrane in migration assay. Table S7. The number of cells passing through the chamber membrane in invasion assay. Table S8. The number of clones formed in the downexpression group and overexpression group. Table S9. The growth size of tumors in nude mice after shRNA transfection. Table S10. The growth size of tumors in nude mice after overexpression transfection. Table S11. The normalized gray value of target genes by positive control points. Table S12. The mRNA levels of PD-L1 in ES-2 cells after shRNA transfection. Table S13. The mRNA levels of PD-L1 in OVCAR-3 cells after overexpression transfection. Table S14. The mRNA levels of PD-L1 in four ovarian cancer cell lines. Table S15. The mRNA levels of PD-L1 in cells. Table S16. The number of cells passing through the chamber membrane in migration or invasion assay. Table S17. The number of clones formed in the downexpression group and overexpression group. Table S18. The growth size of tumors in nude mice after PD-L1 shRNA or cDNA transfection. Table S19. The number of cells passing through the chamber membrane in migration or invasion assay. Table S20. The number of clones formed in the downexpression group and overexpression group. Table S21. The growth size of tumors in nude mice after PD-L1 cDNA transfection [file 11658_2023_471_MOESM1_ESM.doc]

**Table 1. The number of cells passing through the chamber membrane in migration assay**

| Cells | Migration assay cell number (Mean±SD) | *P* |
| --- | --- | --- |
| ES-2 | 498.33±36.91 |  |
| SKOV3 | 317.33±32.02 | 0.003 |
| CAOV-3 | 84.67±13.32 | ＜0.001 |
| OVCAR-3 | 61.33±15.53 | ＜0.001 |

**Table 2. The number of cells passing through the chamber membrane in invasion assay**

| Cells | Invasion assay cell number (Mean±SD) | *P* |
| --- | --- | --- |
| ES-2 | 262.67±22.12 |  |
| SKOV3 | 163.33±20.21 | 0.005 |
| CAOV-3 | 47.00±11.14 | ＜0.001 |
| OVCAR-3 | 13.67±4.04 | ＜0.001 |

**Table 3. The mRNA levels of EFEMP2 in four ovarian cancer cell lines**

| Cells | Relative mRNA level (Mean±SD) | *P* |
| --- | --- | --- |
| OVCAR-3 | 1.00 |  |
| CAOV-3 | 5.52±0.85 | ＜0.001 |
| SKOV3 | 8.56±0.51 | ＜0.001 |
| ES-2 | 14.86±0.35 | ＜0.001 |

**Table 4. The mRNA levels of EFEMP2 in ES-2 cells after shRNA transfection**

| Cells | Relative mRNA level (Mean±SD) | *P* |
| --- | --- | --- |
| ES-2 | 1.00 |  |
| ES-2 NC | 0.99±0.05 | 0.657 |
| ES-2 shRNA1 | 0.19±0.05 | ＜0.001 |
| ES-2 shRNA2 | 0.26±0.10 | ＜0.001 |

**Table 5.** **The mRNA levels of EFEMP2 in OVCAR-3** **cells after overexpression transfection**

| Cells | Relative mRNA level (Mean±SD) | *P* |
| --- | --- | --- |
| OVCAR-3 | 1.00 |  |
| OVCAR-3 NC | 1.04±0.10 | 0.497 |
| OVCAR-3 EX | 15.40±1.35 | ＜0.001 |

**Table 6. The number of cells passing through the chamber membrane in migration assay**

| Cells | Migration assay cell number (Mean±SD) | *P* |
| --- | --- | --- |
| ES-2 NC | 582.67±28.45 |  |
| ES-2 shRNA1 | 120.00±7.00 | ＜0.001 |
| ES-2 shRNA2 | 132.67±11.85 | ＜0.001 |
| OVCAR-3 NC | 75.00±8.54 |  |
| OVCAR-3 EX | 152.33±12.50 | ＜0.001 |

**Table 7. The number of cells passing through the chamber membrane in invasion assay**

| Cells | Invasion assay cell number (Mean±SD) | *P* |
| --- | --- | --- |
| ES-2 NC | 466.00±32.19 |  |
| ES-2 shRNA1 | 61.00±9.85 | ＜0.001 |
| ES-2 shRNA2 | 48.00±9.17 | ＜0.001 |
| OVCAR-3 NC | 27.33±6.66 |  |
| OVCAR-3 EX | 85.33±11.24 | 0.002 |

**Table 8. The number of clones formed in the downexpression group and overexpression group**

| Cells | Number of clones (Mean±SD) | *P* |
| --- | --- | --- |
| ES-2 NC | 569.00±21.93 |  |
| ES-2 shRNA1 | 293.33±18.15 | ＜0.001 |
| ES-2 shRNA2 | 462.33±31.37 | 0.009 |
| OVCAR-3 NC | 250.33±11.50 |  |
| OVCAR-3 EX | 581.33±22.75 | ＜0.001 |

**Table 9. The growth size of tumors in nude mice after shRNA transfection**

| Cells | Tumor volume (mm^3^) (Mean±SD) | *P* |
| --- | --- | --- |
| ES-2 NC | 1655.25±462.16 |  |
| ES-2 shRNA1 | 618.32±173.91 | 0.003 |

**Table 10. The growth size of tumors in nude mice after overexpression transfection**

| Cells | Tumor volume (mm^3^) (Mean±SD) | *P* |
| --- | --- | --- |
| OVCAR-3 NC | 224.39±52.75 |  |
| OVCAR-3 EX | 423.38±105.05 | 0.005 |

**Table 11. The normalized gray value of target genes by positive control points**

| Cells | Gray value (Mean±SD) | *P* |
| --- | --- | --- |
| EGFR |  |  |
| ES-2 NC | 0.91±0.10 |  |
| ES-2 shRNA1 | 0.72±0.02 | 0.032 |
| c-Jun |  |  |
| ES-2 NC | 0.29±0.01 |  |
| ES-2 shRNA1 | 0.12±0.04 | 0.001 |

**Table 12. The mRNA levels of PD-L1 in ES-2 cells after shRNA transfection**

| Cells | Relative mRNA level (Mean±SD) | *P* |
| --- | --- | --- |
| ES-2 | 1.00 |  |
| ES-2 NC | 0.98±0.03 | 0.261 |
| ES-2 shRNA1 | 0.43±0.08 | ＜0.001 |
| ES-2 shRNA2 | 0.54±0.03 | ＜0.001 |

**Table 13. The mRNA levels of PD-L1 in OVCAR-3 cells after overexpression transfection**

| Cells | Relative mRNA level (Mean±SD) | *P* |
| --- | --- | --- |
| OVCAR-3 | 1.00 |  |
| OVCAR-3 NC | 0.98±0.13 | 0.832 |
| OVCAR-3 EX | 1.68±0.10 | ＜0.001 |

**Table 14. The mRNA levels of PD-L1 in four ovarian cancer cell lines**

| Cells | Relative mRNA level (Mean±SD) | *P* |
| --- | --- | --- |
| OVCAR-3 | 1.00 |  |
| CAOV-3 | 4.76±0.75 | 0.001 |
| SKOV3 | 5.80±0.50 | ＜0.001 |
| ES-2 | 21.77±1.60 | ＜0.001 |

**Table 15. The mRNA levels of PD-L1 in cells**

| Cells | Relative mRNA level  (Mean±SD) | *P* |
| --- | --- | --- |
| ES-2 |  |  |
| Non-infected | 1.00 |  |
| NC shRNA | 0.99±0.05 | 0.651 |
| PD-L1 shRNA | 0.45±0.04 | ＜0.001 |
| OVCAR-3 |  |  |
| Non-infected | 1.00 |  |
| NC cDNA | 0.97±0.02 | 0.145 |
| OVCAR-3 PD-L1 cDNA | 22.39±4.35 | 0.020 |
| EFEMP2 cDNA transfected OVCAR-3 |  |  |
| Non-infected | 1.00 |  |
| EFEMP2 cDNA+ NC shRNA | 0.95±0.07 | 0.284 |
| EFEMP2 cDNA+PD-L1 shRNA | 0.30±0.07 | ＜0.001 |
| EFEMP2 shRNA transfected ES-2 |  |  |
| Non-infected | 1.00 |  |
| EFEMP2 shRNA+ NC cDNA | 1.00±0.05 | 0.900 |
| EFEMP2 shRNA+PD-L1 cDNA | 21.15±1.46 | 0.003 |

**Table 16. The number of cells passing through the chamber membrane in migration or invasion assay**

| Cells | Cell number (Mean±SD) | *P* |
| --- | --- | --- |
| Migration assay |  |  |
| ES-2 NC shRNA | 423.33±87.37 |  |
| ES-2 PD-L1 shRNA | 181.00±18.52 | 0.009 |
| OVCAR-3 NC cDNA | 195.00±19.29 |  |
| OVCAR-3 PD-L1 cDNA | 316.67±5.03 | ＜0.001 |
| Invasion assay |  |  |
| ES-2 NC shRNA | 112.00±18.33 |  |
| ES-2 PD-L1 shRNA | 31.33±11.06 | 0.003 |
| OVCAR-3 NC cDNA | 15.67±3.79 |  |
| OVCAR-3 PD-L1 cDNA | 25.00±2.65 | 0.025 |

**Table 17. The number of clones formed in the downexpression group and overexpression group**

| Cells | Number of clones (Mean±SD) | *P* |
| --- | --- | --- |
| ES-2 NC shRNA | 526.33±25.11 |  |
| ES-2 PD-L1 shRNA | 197.00±8.19 | ＜0.001 |
| OVCAR-3 NC cDNA | 479.00±51.64 |  |
| OVCAR-3 PD-L1 cDNA | 716.67±30.44 | 0.002 |

**Table 18. The growth size of tumors in nude mice after PD-L1 shRNA or cDNA transfection**

| Cells | Tumor volume (mm^3^) (Mean±SD) | *P* |
| --- | --- | --- |
| ES-2 NC shRNA | 1257.24±160.92 |  |
| ES-2 PD-L1 shRNA | 296.91±103.27 | ＜0.001 |
| OVCAR-3 NC cDNA | 230.00±41.50 |  |
| OVCAR-3 PD-L1 cDNA | 583.60±136.65 | ＜0.001 |

**Table 19. The number of cells passing through the chamber membrane in migration or invasion assay**

| Cells | Cell number  (Mean±SD) | *P* |
| --- | --- | --- |
| Migration assay |  |  |
| OVCAR-3 EFEMP2 cDNA+ NC shRNA | 221.00±3.61 |  |
| OVCAR-3 EFEMP2 cDNA+PD-L1 shRNA | 61.33±9.61 | ＜0.001 |
| ES-2 EFEMP2 shRNA+ NC cDNA | 93.00±6.25 |  |
| ES-2 EFEMP2 shRNA+PD-L1 cDNA | 180.33±10.50 | ＜0.001 |
| Invasion assay |  |  |
| OVCAR-3 EFEMP2 cDNA+ NC shRNA | 155.00±20.81 |  |
| OVCAR-3 EFEMP2 cDNA+PD-L1 shRNA | 28.33±4.04 | ＜0.001 |
| ES-2 EFEMP2 shRNA+ NC cDNA | 47.00±6.56 |  |
| ES-2 EFEMP2 shRNA+PD-L1 cDNA | 95.33±8.50 | 0.002 |

**Table 20. The number of clones formed in the downexpression group and overexpression group**

| Cells | Number of clones  (Mean±SD) | *P* |
| --- | --- | --- |
| OVCAR-3 EFEMP2 cDNA+ NC shRNA | 590.33±135.69 |  |
| OVCAR-3 EFEMP2 cDNA+PD-L1 shRNA | 213.33±17.21 | 0.009 |
| ES-2 EFEMP2 shRNA+ NC cDNA | 333.00±11.27 |  |
| ES-2 EFEMP2 shRNA+PD-L1 cDNA | 691.33±11.02 | ＜0.001 |

**Table 21. The growth size of tumors in nude mice after PD-L1 cDNA transfection**

| Cells | Tumor volume (mm^3^)  (Mean±SD) | *P* |
| --- | --- | --- |
| ES-2 EFEMP2 shRNA+ NC cDNA | 449.00±86.10 |  |
| ES-2 EFEMP2 shRNA+PD-L1 cDNA | 990.00±274.59 | 0.003 |
| OVCAR-3 EFEMP2 cDNA+ NC shRNA | 512.80±148.89 |  |
| OVCAR-3 EFEMP2 cDNA+PD-L1 shRNA | 112.00±20.08 | ＜0.001 |
